# Circulating vitamin C and digestive system cancers: Mendelian randomization study

**DOI:** 10.1016/j.clnu.2022.07.040

**Published:** 2022-08-07

**Authors:** Susanna C. Larsson, Amy M. Mason, Mathew Vithayathil, Paul Carter, Siddhartha Kar, Ju-Sheng Zheng, Stephen Burgess

**Affiliations:** a Unit of Medical Epidemiology, Department of Surgical Sciences, Uppsala University, Uppsala, Sweden; b Unit of Cardiovascular and Nutritional Epidemiology, Institute of Environmental Medicine, Karolinska Institutet, Stockholm, Sweden; c British Heart Foundation Cardiovascular Epidemiology Unit, Department of Public Health and Primary Care, University of Cambridge, Cambridge, United Kingdom; d National Institute for Health Research Cambridge Biomedical Research Centre, University of Cambridge and Cambridge University Hospitals, Cambridge, United Kingdom; e MRC Cancer Unit, University of Cambridge, Cambridge, United Kingdom; f Department of Medicine, University of Cambridge, Cambridge, United Kingdom; g MRC Integrative Epidemiology Unit, Bristol Medical School, University of Bristol, Bristol, United Kingdom; h Key Laboratory of Growth Regulation and Translational Research of Zhejiang Province, School of Life Sciences, Westlake University, Hangzhou, China; i Westlake Laboratory of Life Sciences and Biomedicine, Hangzhou, China; j Institute of Basic Medical Sciences, Westlake Institute for Advanced Study, Hangzhou, China; k Department of Public Health and Primary Care, University of Cambridge, Cambridge, United Kingdom; l MRC Biostatistics Unit, University of Cambridge, Cambridge, United Kingdom

**Keywords:** ascorbic acid, cancer, digestive system, Mendelian randomization, nutrients, vitamin C

## Abstract

**Background & aims:**

Vitamin C is an antioxidant with a potential role in the prevention of digestive system cancers, but there is yet no consensus whether vitamin C has a causal role in these cancers. The aim of this study was to utilize Mendelian randomization to decipher the potential causal associations of vitamin C with risk of digestive system cancers.

**Methods:**

Ten genetic variants previously found to be significantly associated with circulating vitamin C were used as instrumental variables. Effect size estimates for the genetic associations of the vitamin C-associated genetic variants with six major malignancies of digestive system were obtained from the FinnGen (N=309 154) and UK Biobank (N=367 542) studies. Results from the two studies were combined using meta-analysis.

**Results:**

Genetically predicted higher circulating vitamin C showed a suggestive association with lower risk of small intestine and colorectal cancer after accounting for multiple testing. The odds ratio per 1 standard deviation increment in circulating vitamin C was 0.55 (95% confidence interval 0.32-0.94; *P*=0.029) for small intestine cancer and 0.84 (95% confidence interval 0.73-0.96; *P*=0.013) for colorectal cancer. There was a suggestive association between genetically predicted higher circulating vitamin C with lower risk of liver cancer in FinnGen but no association in the meta-analysis (odds ratio 0.69; 95% CI 0.36-1.32; *P*=0.265). Genetically predicted circulating vitamin C was not associated with cancers of the esophagus, stomach, or pancreas.

**Conclusion:**

This Mendelian randomization study indicates that vitamin C might play a role in the prevention of small intestine and colorectal cancer.

## 1 Introduction

Vitamin C, also known as ascorbic acid, is an essential water-soluble nutrient present in most fruits, berries, and vegetables. Most of its biological functions involve its capacity to act as an antioxidant and a free radical scavenger [1–4]. Vitamin C also has a role in the formation of collagen, immune defense system, biosynthesis of certain neurotransmitters, and cell signalling pathways [1–5]. Evidence indicates that vitamin C may play a role in the prevention of cancer, particularly digestive system cancers [1, 2]. Case-control and cohort studies have suggested possible protective associations of a high vitamin C intake with reduced risks of cancers of the digestive system overall [6], colorectum [7, 8], stomach [9, 10], and pancreas [11]. Limited data from observational studies are also suggestive of inverse associations between consumption of vitamin C rich foods, citrus fruits, and vegetables and risk of colon, stomach, and esophageal cancer, respectively [12]. However, residual confounding from other dietary and lifestyle factors is inevitable in observational studies. Furthermore, dietary intake is challenging to measure, leading to misclassification of vitamin C intake and attenuated results.

Randomized controlled trials (RCT), where participants are randomized to receive either vitamin C supplement or placebo, can minimize confounding and could assist in establishing the causal role of vitamin C in cancer prevention. Nevertheless, RCTs of cancer are costly and impractical to execute due to several reasons, such as large sample sizes and long follow up required as cancer cells can take decades to develop. Hitherto, only a few RCTs have reported results on the effect of vitamin C supplementation on digestive system cancers and those trials included few incident cancer cases and found no significant associations (Table 1) [13–17], albeit results for colorectal cancer were suggestive of a protective effect [16, 17].

Mendelian randomization (MR) exploits genetic variants as instrumental variables for modifiable exposures to infer potential causal associations between exposures and outcomes [18, 19]. The MR design shares similarities to an RCT due to the random allocation of genetic variants when passed from parents to offspring and are therefore generally independent of confounders. Here, we performed a two-sample MR study to investigate the causal associations of lifelong higher circulating vitamin C levels with risk of major malignancies of the digestive system.

## 2 Materials and Methods

### 2.1 Genetic Instrument Selection

We obtained genetic instruments from the hitherto largest genome-wide association meta-analysis of circulating vitamin C which included 52 018 individuals of European ancestries from the Fenland study, European Prospective Investigation into Cancer and Nutrition (EPIC)-InterAct study, EPIC Norfolk study, and EPIC-CVD study [7]. That meta-analysis identified 11 single-nucleotide polymorphisms (SNPs) associated with plasma vitamin C at the genome-wide significance threshold (*P*<5×10^-8^) after adjustment for age, sex, ten genetic principal components, and study center [7]. The SNPs explained ~1.9% of the variance in plasma vitamin C.[7] One of the SNPs (rs174547) is located in the *FADS1* gene which encodes the rate-limiting enzyme in polyunsaturated fatty acid synthesis and is significantly associated with plasma phospholipid arachidonic acid [20]. This SNP was excluded from our MR analyses as genetically predicted plasma phospholipid arachidonic acid is associated with certain cancers, including colorectal, esophageal, and lung cancers [20]. The remaining ten SNPs were used as instrumental variables in this MR study (Table 2).

### 2.2 Data Sources for Cancer Outcomes

We obtained effect size estimates for the genetic associations of the vitamin C-associated SNPs with digestive system cancer from the FinnGen and UK Biobank studies. Malignant neoplasms of the digestive system with at least 300 cases in each study (i.e., cancers of the esophagus, stomach, small intestine, colorectum, pancreas, and liver) were included. For FinnGen, we used the latest publicly available data release (freeze 7) which included 309 154 men and women [21]. One of the vitamin C-associated SNPs (rs13028225) was unavailable in FinnGen for which a proxy variant (rs17655123) in high linkage disequilibrium (R^2^=0.93 in the Finnish population) with the specified SNP was used. For UK Biobank, we estimated the genetic associations of the vitamin C-associated SNPs with the digestive system cancers in 367 542 unrelated participants (37-73 years of age at the baseline assessment) of European ancestries using logistic regression, adjusted for age and the first ten genetic principal components, as previously described [22]. Cancer outcomes were classified according to data from the national cancer registry, electronic health records, and hospital episode statistics and death certificates and verified self-reported information. The cancer registry censor dates were July 31, 2019, for England and Wales and October 31, 2015, for Scotland. The hospital episode statistics and self-reported data was downloaded on July 27, 2021, with censoring dates defined by UK Biobank at that time as March 31, 2021, for English hospital data, February 28, 2018, for Wales Hospital data, and March 31, 2021, for Scottish hospital data. Death records data were available up to 28 February 2021. Classifications of cancers in the FinnGen and UK Biobank studies are shown in Table S1. The FinnGen and UK Biobank were approved by a relevant ethical review board, and participants provided informed consent. The MR analyses were approved by the Swedish Ethical Review Authority (No. 2019-02793).

### 2.3 Statistical Analysis

The multiplicative random-effects inverse variance weighted (IVW), weighted median, and MR-Egger regression methods were applied as statistical methods in this MR study [23]. These three approaches have different assumptions, as described in detail elsewhere [23]. The IVW method provides estimates with the greatest precision and was considered the main analysis, whereas the other two methods were used as sensitivity analyses to assess the robustness of the results. All odds ratios (OR) with corresponding 95% confidence intervals (CI) were scaled per 1 standard deviation increase in circulating vitamin C levels (standardized residuals in standard deviation units were calculated in the original genome-wide association study of vitamin C [7]). Associations were considered statistically significant at the Bonferroni-corrected threshold of *P*<0.008 (*P*=0.05/6 outcomes), and suggestive at *P* values between 0.008 and 0.05. Stata (StataCorp, College Station, Texas) and R statistical software were used to analyze the data and construct the figures. For the MR analyses, the mrrobust command for Stata was used. The metan command for meta-analysis in Stata was used to combine estimate from the two studies for each digestive system cancer. Between-study heterogeneity was assessed by the *I*^2^ statistic [24].

## 3 Results

**Fig. 1:**
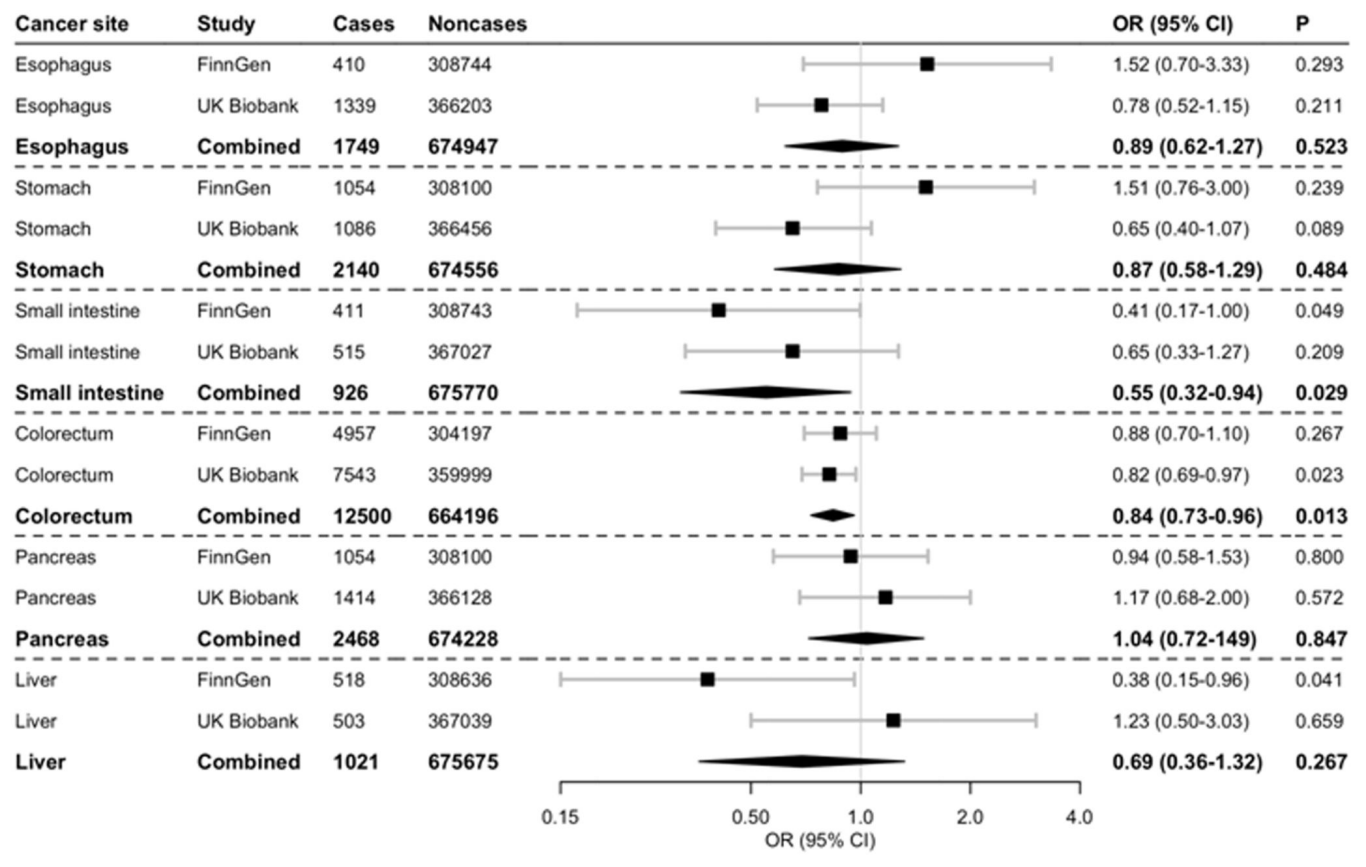
Associations of genetically predicted circulating vitamin C with digestive system cancers in FinnGen and UK Biobank and in meta-analysis of both studies. The odds ratios are scaled per 1 standard deviation increase in circulating vitamin C. Abbreviations: CI, confidence interval; OR, odds ratio.

Genetically predicted circulating vitamin C was non-significantly associated with lower risk of small intestine and colorectal cancer at the Bonferroni-corrected significance threshold (Fig. 1, Fig. S1, and Fig. S2). In the meta-analysis of the two studies, the OR per 1 standard deviation increment in circulating vitamin C was 0.55 (95% CI 0.32-0.94; *P*=0.029) for small intestine cancer and 0.84 (95% CI 0.73-0.96; *P*=0.013) for colorectal cancer (Fig. 1). The association appeared stronger for colon cancer (OR 0.79; 95 CI 0.66-0.94; *P*=0.009) than for rectal cancer (OR 0.89; 95 CI 0.70-1.12; *P*=0.312) but the difference was not statistically significant (*P*=0.427). Genetically predicted higher circulating vitamin C was non-significantly associated with a lower risk of liver cancer in FinnGen but there was no overall association in the meta-analysis of the two studies (OR 0.69; 95% CI 0.36-1.32; *P*=0.265) (Fig. 1). No association was observed between genetically predicted circulating vitamin C and cancers of the esophagus, stomach, and pancreas (Fig. 1). Moderate to substantial heterogeneity between results of the two studies was found in the analyses of esophageal (*I*^2^=56%), stomach (*I*^2^=74%), and liver (*I*^2^=68%) cancer, but there was no between-study heterogeneity in the analyses of the other cancer sites (*I*^2^=0%). Sensitivity analyses revealed similar result, albeit of lower precision, and there was no evidence of directional pleiotropy (Table S2).

## 4 Discussion

This MR study aimed at deciphering the potential causal role of vitamin C in the prevention of digestive system cancers by using data from two large studies of European populations. Results showed evidence of suggestive inverse associations of genetically predicted circulating vitamin C with lower risk of small intestine and colorectal cancer. There was no overall association of genetically predicted circulating vitamin C with the other four studied digestive system cancers, but a suggestive inverse association was found for liver cancer in the FinnGen study.

### 4.1 Vitamin C and small intestine cancer

Our MR finding of a suggestive inverse association between circulating vitamin C and risk of small intestine cancer in two independent populations was novel. The etiology of small intestine cancer remains largely unknown, but the present study provides some evidence that vitamin C may have a preventive role in this rare cancer form. Further studies of vitamin C, antioxidants, and oxidative stress in relation to risk of small intestine cancer is warranted.

### 4.2 Vitamin C and colorectal cancer

Our finding for circulating vitamin C in relation to risk of colorectal cancer corroborates and extends the results of previous studies. A recent meta-analysis of prospective cohort studies showed that high intakes of dietary vitamin C (4 studies), supplemental vitamin C (2 studies), and dietary and supplemental vitamin C (1 study) were non-significantly associated with lower risk of colorectal cancer (relative risks of 0.86, 0.80 and 0.60, respectively, for the highest versus lowest exposure category) [7]. A meta-analysis of two RCTs [16, 17] (Table 1) also suggested a reduction in colorectal cancer incidence in participants who received vitamin C supplements compared with the placebo group, but the precision of the estimate was low due to few cancer cases (relative risk 0.84, 95% CI 0.64-1.10) [7]. Likewise, genetically predicted higher circulating vitamin C levels (per 1 standard deviation increment) were non-significantly associated with 15-21% lower risk of colon cancer (n=3221 cases) in a previous MR analysis based on the UK Biobank study [7]. Our MR study, which included a larger number of cases, extends earlier evidence by showing a suggestive association of circulating vitamin C with colorectal cancer risk in a combined analysis of the FinnGen and UK Biobank studies with a total of 12 500 colorectal cancer cases. Experimental studies have shown that supplementation with vitamin C reduces proliferation of adenomatous colonic polyps (precursors to colorectal cancer) [25] and that the anti-proliferative effects may be mediated through selective impairment of growth in KRAS and BRAF mutant cells that are frequently seen in colorectal cancers [26].

### 4.3 Strengths and limitations

An important advantage of this investigation is that results were derived using MR, a method that minimizes confounding and reverse causation bias. The genetic instrument for circulating vitamin C has been shown to be unrelated to fruit and vegetable intake, lifestyle factors (e.g., alcohol consumption, physical activity, smoking, and vitamin supplement use), body mass index, and education [7]. The SNPs applied as instrumental variables were located in genes involved in direct transport or regulation of vitamin C concentration (e.g., vitamin C absorption, renal reabsorption, and cellular uptake) or in genes associated with antioxidant and oxidative stress [7]. The SNP (rs33972313) in *SLC23A1*, which has the strongest association with plasma vitamin C, encodes a sodium-dependent vitamin C transporter [7]. The *SLC23A1* variant had a strong association with small intestine cancer (Figure S1) and colorectal cancer (Figure S2) in both FinnGen and UK Biobank. Another strength is that we investigated the associations of circulating vitamin C with several digestive system cancers and utilized genome-wide association study data from two large studies, thereby improving statistical power. Furthermore, weak instrument bias was minimized as there was no sample overlap between the exposure and outcome datasets and the strength of the vitamin C genetic instrument was high (F statistic of 30.5) [7].

A possible shortcoming of this MR study is that the exposure and outcome datasets only included individuals of European descents only, thereby confining the generalizability of our results. However, this restriction minimized possible bias due to population stratification. This study is limited by the relatively few cases of esophageal, stomach, small intestine, pancreas, and liver cancer. We therefore cannot rule out weak associations of circulating vitamin C with other digestive system cancers.

## 5 Conclusions

This MR study based on data from two large studies comprising Finnish and British populations found evidence to support a possible protective association of higher circulating vitamin C with small intestine cancer and colorectal cancer. The MR finding for small intestine cancer was novel and needs validation in future studies. The observed finding for colorectal cancer in this MR study along with previous suggestive associations in prospective cohort studies and RCTs triangulate the evidence that increasing circulating vitamin C levels through high consumption of vitamin C-rich foods or supplementation may reduce the risk of colorectal cancer.

## Supplementary Material

Supplementary materials

## Figures and Tables

**Table 1:** Randomized controlled trials with results on the effect of vitamin C supplements, with or without other micronutrients, on digestive system cancer*

				No. of incident cases			
Trial name; average follow-up time	Daily supplement	Sample size	Cancer site	Supplement group	Placebo group	RR	95% CI
Linxian Trial [13]; 6 yrs	Vitamin C 180 mg plus 25 other micronutrients	3318†	Stomach	96	81	1.17	0.73-1.20
		3318†	Esophagus	123	128	0.94	0.87-1.58
MRC/BHF Heart Protection Study [14]; 5 yrs	Vitamin C 250 mg, vitamin E 600 mg, β-carotene 20 mg	20 536	Digestive tract	228	223	NA	NA
SU.VI.MAX [15]; 7.5 yrs	Vitamin C 120 mg, vitamin E 30 mg, β-carotene 6 mg, selenium 100 μg, zinc 20 mg	13 017	Digestive tract	18/15‡	25/15‡	NA	NA
Physicians’ Health Study II [16]; 7.6 yrs	Vitamin C 500 mg, vitamin E 400 IU, β-carotene 50 mg	14 520	Colorectum	75	87	0.86	0.63-1.17
		14 638	Pancreas	27	28	0.97	0.57-1.64
Women’s Antioxidant Cardiovascular Study [17]; 9.4 yrs	Vitamin C 500 mg, vitamin E 600 IU, β-carotene 50 mg	8171	Colorectum	19	25	0.76	0.42-1.38
		8171	Pancreas	14	6	2.32	0.89-6.04

Abbreviations: CI, confidence interval; RR, relative risk (rate ratio or hazard ratio).

* Only results for digestive system cancers are presented herein but most trials also provided results on overall cancer, non-digestive system cancers, cardiovascular disease, and all-cause mortality.

† Patients with esophageal dysplasia.

‡ Number of cancer cases among men/women.

**Table 2:** Genetic variants used as instrumental variables for circulating vitamin C levels

SNP	Chr	Nearby gene	EA	OA	EAF	Beta*	SE*	*P*
rs6693447	1	*RER1*	T	G	0.551	0.039	0.006	6.25E-10
rs13028225	2	*SLC23A3*	T	C	0.857	0.102	0.009	2.38E-30
rs33972313	5	*SLC23A1*	C	T	0.968	0.360	0.018	4.61E-90
rs10051765	5	*RGS14*	C	T	0.342	0.039	0.007	3.64E-09
rs7740812	6	*GSTA5*	G	A	0.594	0.038	0.006	1.88E-09
rs117885456	12	*SNRPF*	A	G	0.087	0.078	0.012	1.70E-11
rs2559850	12	*CHPTI*	A	G	0.598	0.058	0.006	6.30E-20
rs10136000	14	*AKTI*	A	G	0.283	0.040	0.007	1.33E-08
rs56738967	16	*MAF*	C	G	0.321	0.041	0.007	7.62E-10
rs9895661	17	*BCAS3*	T	C	0.817	0.063	0.008	1.05E-14

Abbreviations: Chr, chromosome; EA, effect allele; EAF, effect allele frequency; OA, other allele; SE, standard error; SNP, single-nucleotide polymorphism.

* Beta coefficients and standard errors represent the increase in plasma vitamin C in standard deviation units per additional effect allele.
