# Supplementary material for: Circulating vitamin C and digestive system cancers: Mendelian randomization study
Source: Clin Nutr. Author manuscript; Available in PMC 2022 Aug 31. (PMC7613472; doi:10.1016/j.clnu.2022.07.040)
Supplement: Supplementary materials [file EMS153059-supplement-Supplementary_materials.docx]

**Supplemental Material**

**Circulating vitamin C and digestive system cancers: Mendelian randomization study**

| ***Content*** | ***Page*** |
| --- | --- |
| **Table S1.** Definitions of each digestive system cancer in the FinnGen and UK Biobank | 2 |
| **Table S2.** Associations of genetically predicted vitamin C with risk of digestive system cancers in sensitivity analyses based on the weighted median and MR-Egger regression methods | 3 |
| **Figure S1.** Scatter plot of the associations of the vitamin C-associated SNPs with circulating vitamin C levels and small intestine cancer in FinnGen and UK Biobank | 4 |
| **Figure S2.** Scatter plot of the associations of the vitamin C-associated SNPs with circulating vitamin C levels and colorectal cancer in FinnGen and UK Biobank | 5 |

**Table S1.** Classification of each digestive system cancer in the FinnGen and UK Biobank

|  | **FinnGen** | |  | **UK Biobank** | | | |
| --- | --- | --- | --- | --- | --- | --- | --- |
| **Cancer site** | **ICD-9 codes** | **ICD-10 codes** |  | **ICD-9 codes** | **ICD-10 codes** | **Self-report (field 20001)*** | **Cancer histology*** |
| Esophagus | 150 | C15 |  | 150, V10.03 | C15, Z85.01 | 1017 |  |
| Stomach | 151 | C16 |  | 151, V10.04 | C16, Z85.028 | 1018 |  |
| Small intestine | 152 | C17 |  | 152 | C17 | 1019 |  |
| Colorectum | 153, 154 | C18, C19, C20 |  | 153, 154.0, 154.1, V10.05, V10.06 | C18, C19, C20, Z85.038, Z85.048 | 1020, 1022, 1023 |  |
| Pancreas | 157 | C25 |  | 157 | C25, Z85.07 | 1026 |  |
| Liver | 155 | C22 |  | 155.0 | C22.0 | 1024 | 8170, 8171, 8172, 8173, 8174, 8175 |

Abbreviations: ICD, international classification of diseases.

*The self-report and cancer histology columns provide the internal UK Biobank codes used to define each outcome (available at <https://biobank.ctsu.ox.ac.uk/crystal/coding.cgi?id=3> and https://biobank.ctsu.ox.ac.uk/crystal/coding.cgi?id=38).

**Table S2.** Associations of genetically predicted vitamin C with risk of digestive system cancers in sensitivity analyses based on the weighted median and MR-Egger regression methods*

|  |  |  |  | **Weighted median** | | | |  | **MR-Egger regression†** | | | |
| --- | --- | --- | --- | --- | --- | --- | --- | --- | --- | --- | --- | --- |
| **Cancer site** | **Study** | **Cases** |  | **OR** | **LB** | **UB** | **P** |  | **OR** | **LB** | **UB** | **P** |
| Esophagus | FinnGen | 410 |  | 1.82 | 0.58 | 5.72 | 0.306 |  | 2.45 | 0.63 | 9.49 | 0.231 |
| Esophagus | UK Biobank | 1339 |  | 0.68 | 0.41 | 1.12 | 0.128 |  | 0.69 | 0.37 | 1.30 | 0.251 |
| Esophagus | Meta-analysis | 1749 |  | 0.80 | 0.50 | 1.26 | 0.334 |  | 0.86 | 0.49 | 1.53 | 0.614 |
| Stomach | FinnGen | 1054 |  | 1.09 | 0.57 | 2.11 | 0.792 |  | 0.99 | 0.30 | 3.31 | 0.991 |
| Stomach | UK Biobank | 1086 |  | 0.70 | 0.40 | 1.23 | 0.211 |  | 0.72 | 0.32 | 1.62 | 0.425 |
| Stomach | Meta-analysis | 2140 |  | 0.84 | 0.55 | 1.29 | 0.437 |  | 0.80 | 0.41 | 1.56 | 0.506 |
| Small intestine | FinnGen | 411 |  | 0.44 | 0.16 | 1.21 | 0.113 |  | 0.34 | 0.07 | 1.78 | 0.239 |
| Small intestine | UK Biobank | 515 |  | 0.67 | 0.30 | 1.51 | 0.330 |  | 0.67 | 0.22 | 2.07 | 0.491 |
| Small intestine | Meta-analysis | 926 |  | 0.57 | 0.30 | 1.07 | 0.080 |  | 0.54 | 0.22 | 1.37 | 0.195 |
| Colorectum | FinnGen | 4957 |  | 0.81 | 0.60 | 1.11 | 0.187 |  | 0.75 | 0.51 | 1.12 | 0.195 |
| Colorectum | UK Biobank | 7543 |  | 0.85 | 0.68 | 1.06 | 0.150 |  | 0.92 | 0.71 | 1.21 | 0.562 |
| Colorectum | Meta-analysis | 12500 |  | 0.84 | 0.70 | 1.00 | 0.053 |  | 0.86 | 0.69 | 1.08 | 0.193 |
| Pancreas | FinnGen | 1054 |  | 1.04 | 0.56 | 1.94 | 0.893 |  | 0.96 | 0.41 | 2.23 | 0.927 |
| Pancreas | UK Biobank | 1414 |  | 1.59 | 0.92 | 2.73 | 0.095 |  | 1.35 | 0.55 | 3.27 | 0.511 |
| Pancreas | Meta-analysis | 2468 |  | 1.32 | 0.88 | 1.99 | 0.179 |  | 1.13 | 0.61 | 2.08 | 0.700 |
| Liver | FinnGen | 518 |  | 0.42 | 0.16 | 1.06 | 0.067 |  | 0.33 | 0.06 | 1.81 | 0.238 |
| Liver | UK Biobank | 503 |  | 1.09 | 0.48 | 2.46 | 0.844 |  | 1.10 | 0.24 | 4.96 | 0.903 |
| Liver | Meta-analysis | 1021 |  | 0.72 | 0.39 | 1.33 | 0.292 |  | 0.65 | 0.21 | 2.01 | 0.450 |

Abbreviations: LB, lower bound of the 95% confidence interval; OR, odds ratio; UB, upper bound of the 95% confidence interval.

*The estimates (OR, LB, and UB) are scaled per 1 standard deviation increase in genetically predicted circulating vitamin C levels.

†There was no evidence of directional pleiotropy, tested by the intercept in the MR-Egger analysis, in any analysis (all *P*>0.25).

**FinnGen**


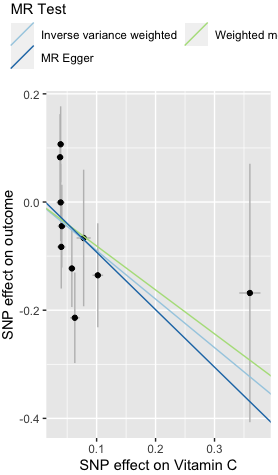


**UK Biobank**

**
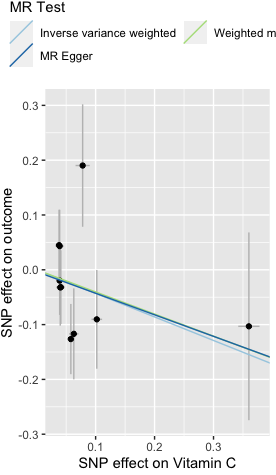
**

**Figure S1.** Scatter plot of the associations of the vitamin C-associated SNPs with circulating vitamin C levels and small intestine cancer in FinnGen and UK Biobank. Abbreviations: MR, Mendelian randomization; SNP, single-nucleotide polymorphism.

**FinnGen**

**
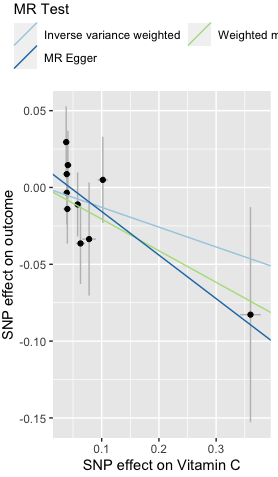
**

**UK Biobank**


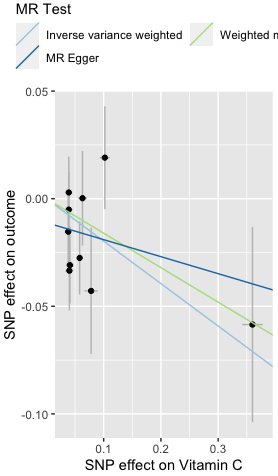


**Figure S2.** Scatter plot of the associations of the vitamin C-associated SNPs with circulating vitamin C levels and colorectal cancer in FinnGen and UK Biobank. Abbreviations: MR, Mendelian randomization; SNP, single-nucleotide polymorphism.
